# Effects of Musts from Esca-Proper-Affected ‘Primitivo’ Vines on Two Commercial *Saccharomyces cerevisiae* Strains

**DOI:** 10.3390/plants15142187

**Published:** 2026-07-16

**Authors:** Giovanni Luigi Bruno, Aurora Acca, Leonardo Scarano, Francesco Mazzone, Francesco Mannerucci, Margherita D’Amico, Marco Vendemia, Antonio Domenico Marsico

**Affiliations:** 1 Department of Soil, Plant and Food Sciences (Di.S.S.P.A.), University of Bari Aldo Moro, Campus “E. Quagliarello”, Via G. Amendola 165/A, 70126 Bari, Italy; auroraacca98@gmail.com (A.A.); francesco.mannerucci@uniba.it (F.M.); 2 Council for Agricultural Research and Economics, Research Centre Viticulture and Enology (CREA-VE), Via Casamassima 148, 70010 Turi, Italy; leonarscara@gmail.com (L.S.); francesco.mazzone@crea.gov.it (F.M.); margherita.damico@crea.gov.it (M.D.); marcovendemia@gmail.com (M.V.); adomenico.marsico@crea.gov.it (A.D.M.)

**Keywords:** *Vitis vinifera*, brown streaks, woody mycobiota, alcoholic fermentation, yeast starter, vinification, grapevine trunk diseases

## Abstract

The grapevine syndrome, Esca proper, causes significant yield losses. This study analyses and discusses the enological traits of musts from healthy (HV) and Esca-proper-affected (EpV) ‘Primitivo’ vines cropped in southern Italy. Tiger-striped leaves, sudden wilting on arms or shoots, berry spots, shrivelling and wilting, wood browning and white rot are linked to the presence of *Phaeoacremonium minimum*, *Phaeomoniella chlamydospora*, and *Fomitiporia mediterranea* within the woody tissues and characterize EpVs. The lack of symptoms on trunks, leaves, berries, and in the wood of branches and shoots, along with the absence of Esca-proper mycota, typifies HVs. Must from the selected HVs is darker and contains greater total soluble solids, reducing sugars, titratable and volatile acidity, tartaric, citric, and L-malic acid, and total polyphenols. The must from the selected EpVs has a higher pH and laccase activity, and contains pullulan, scytalone, and isosclerone, secondary metabolites of *P. minimum* and *P. chlamydospora*. During fermentation, musts from EpVs negatively impact the growth and enological performance of *Saccharomyces cerevisiae* Byosal HS1™ and IOC 18-2007™ and reduce the overall quality of the resulting wines. The physical–chemical conditions during fermentation may degrade pullulan and aid scytalone oxidation to isosclerone.

## 1. Introduction

The so-called ‘Esca proper’, one of the oldest and most damaging diseases affecting grapevine (*Vitis vinifera*) worldwide, includes wood streaking and discolouration, grapevine leaf stripe disease (GLSD), black measles (dark purple spots) on berries, which often shrivel and wilt, and wood white rot [1,2,3]. Apoplectic symptoms (sudden wilting) on either the whole plant or individual arms also commonly occur during the hot summer period [3,4,5,6,7]. Drought and heat stress, macro- and micro-nutrient imbalances in plants, disrupted vine physiology, pathogens’ aggressiveness, and their interactions all contribute to Esca proper development [2,3,4,5]. These physiological alterations affect tiger stripes, black measles, and apoplexy appearance, progression, and year-to-year appearance/absence [7,8,9,10,11,12,13,14,15,16,17]. *Phaeomoniella chlamydospora* and *Phaeoacremonium minimum*, with different *Phaeoacremonium* and *Cadophora* species, trigger brown wood streaking and GLSD [2,8,18,19]. *Fomitiporia mediterranea* and *F. punctata* cause white rot in the Mediterranean region and Central and Northern Europe, respectively [2,20,21]. Leaf and berry symptoms lack pathogen presence but are linked to cultivar susceptibility, vine age, wood-colonizing microorganisms, pedoclimatic conditions, and plants’ physiological factors [3,6,9,17,22,23,24,25,26].

Several secondary metabolites are produced in liquid cultures by *F. mediterranea* [27,28], *P. minimum* [27,29,30,31,32], and *P. chlamydospora* [27,29,30,31,32] and display biological activity toward grapevine leaves, calli, and living protoplasts [27,28,29,30,31,32,33,34]. Finally, pullulan, scytalone, and isosclerone, produced in vitro and in planta by *P. minimum* and *P. chlamydospora* [27,29,30,31], influence the growth and enological performance of the commercial *Saccharomyces cerevisiae* strains Byosal HS1™ and IOC 18-2007™ in a synthetic must [35].

Tannase, laccase, peroxidases, plant-cell-wall-degrading enzymes [36,37,38], reactive hydroxyl radicals [39], phytotoxins [27,28,29,30,31,33], and extracellular proteins [32] allow *P. minimum*, *P. chlamydospora*, and *F. mediterranea* to develop wood streaking or white rot.

Esca-proper-affected plants exhibit physiological alterations [4,40,41,42,43,44,45,46,47,48,49]. These plants reduce the yields of grapes, musts, and ethanol, shorten vine lifespan, increase trans-resveratrol levels in berries and wines, and increase total acidity in the must [49,50,51,52,53,54], thereby influencing wine quality and sensory attributes [3,10,12,22,48,49,50,51,52,53,54,55].

This paper investigates the impact of Esca-proper-affected status on (i) the chemistry of cv ‘Primitivo’ musts, (ii) the growth and enological performance of the *S. cerevisiae* commercial strains Byosal HS1™ and IOC 18-2007™, and (iii) the composition of young wine. These two strains were selected for their ability to ferment various grape juices and synthetic musts alone or synthetic musts amended with pullulan, scytalone, and isosclerone [35]. They avoid the production of undesirable compounds, show resistance to ethanol and sulphur dioxide, are effective at a low pH, and have an 8–30 °C temperature range (https://www.enartis.com; https://ioc.eu.com, accessed on 20 May 2026). The experimental design was intentionally conceived to achieve this objective under realistic vineyard conditions. Special attention is given to pullulan, scytalone, and isosclerone in musts and wine. The analysis of variance (ANOVA) was utilized to examine the relationship between disease occurrence in plants and the composition of musts and wine.

## 2. Results

### 2.1. Vine Selection

**Figure 1:**
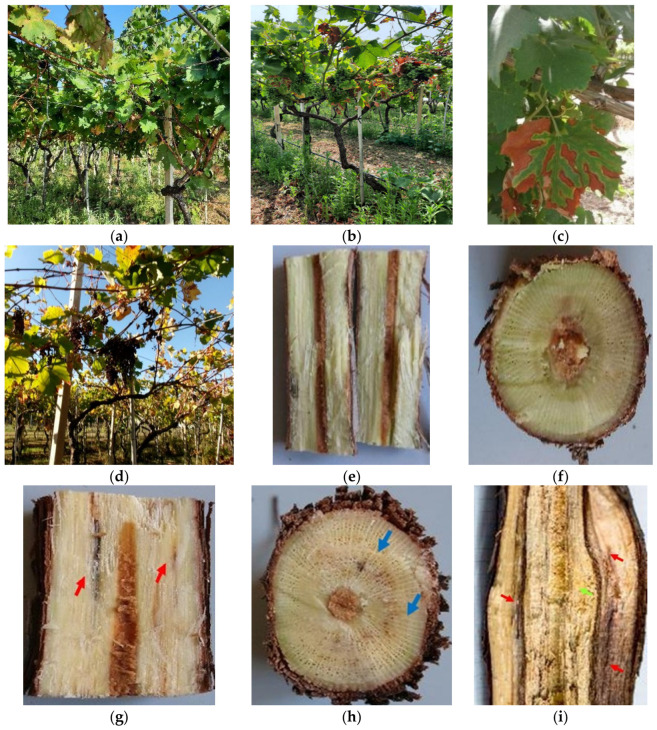
Representative examples of healthy (**a**,**e**,**f**) and Esca-proper-affected (**b**,**g**–**i**) vines of 20-year-old *V. vinifera* cv Primitivo in the vineyard: black measles (**b**), tiger stripes (**b**,**c**), and apoplexy (**d**); longitudinal (**e**,**g**,**i**) and cross (**f**,**h**) sections of wood. Arrows indicate brown streaks (red), black spots (blue), and white rot (green). The pictures show the appearance in July (**a**–**d**) and November (**e**–**i**) 2022.

Out of the 50 ‘Primitivo’ vines surveyed during the 2018–2022 vegetative growing season, 20 (40% of the total plants) always exhibited an asymptomatic canopy and trunk (Figure 1a). Tiger stripes (Figure 1b,c) were observed and documented on 30 vines in 2018, 2020, and 2022, and on 27 vines during the growing cycles of 2019, 2021, and 2023. Apoplexy (Figure 1d) appeared on branches or shoots from two, three, one, and eight vines in the years 2018, 2020, 2021, and 2022, respectively. Crack symptoms in the main trunk were noted on 12 vines during the survey. Black measles during the pre-veraison phenological stage, as well as berry shrivelling and wilting, developed on five, three, one, and 10 vines throughout the 2018, 2020, 2021, and 2022 growth periods. Wood browning and white rot in branches and shoots (Figure 1e–i) further differentiated Esca-proper-affected (EpV) from healthy (HV) vines.

The isolation procedure allowed for the development of more than 180 colonies (Table 1). From brown wood, *P. minimum* and *P. chlamydospora* were isolated, while *F. mediterranea* was obtained from wood showing white rot. Based on these results and considering the number of times the symptoms appeared during the survey period, among the 50 available vines—excluding the most external plants and those with complete apoplexy—five symptomless plants that did not show any foliar symptoms or wood alterations and five with brown wood streaking and white rot were selected.

These plants were used in September 2023 and 2024 for grape harvest and must production. The five EpVs during the 2023 vegetative growing season exhibited crack symptoms in the main trunk, tiger stripes, apoplexy on some shoots, and black measles, berry shrivelling, and wilting. During the 2024 vegetative growing season, the chosen HVs and EpVs did not exhibit any tiger stripes, black measles, or signs of apoplexy. Of the other vines, 15 showed asymptomatic canopy and trunk, and seven displayed tiger stripes and cracks on the main trunk. At the same time, 13 developed apoplexies on branches or shoots, black measles, berry shrivelling, or wilting.

### 2.2. Must Characterization

**Figure 2:**
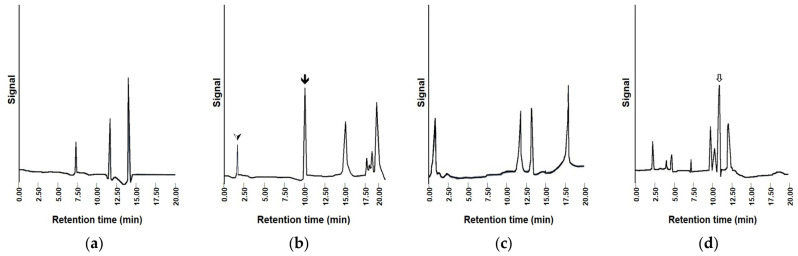
Separation by high-resolution liquid chromatography (HPLC) of 50 (**a**) and 5 (**b**) μg ethyl acetate extracts, and 100 (**c**) or 4 (**d**) μg of ethanol precipitates washed with methanol of musts obtained from healthy (**a**,**c**) or Esca-proper-affected (**b**,**d**) *V. vinifera* cv Primitivo berries. Symbols indicate scytalone (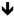), isosclerone (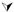), and pullulan (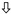). Data refer to the 2023 must collection.

The characteristics of musts obtained from the bunches of the two selected vine groups in 2023 and 2024 are depicted in Table 2 and Figure 2. Despite the absence of symptoms in 2024, the characteristics of the musts show no relevant differences from those produced in 2023. In all measurements, values for musts from HVs were consistently greater than those recorded for EpVs (Table 2). Pullulan, scytalone, and isosclerone were found exclusively in musts obtained from EpVs (Table 2, Figure 2). The enological characteristics of must were not affected by the sampling years but were strongly linked to vine typology (Supplementary Table S1).

### 2.3. Microfermentation Experiments

**Figure 3:**
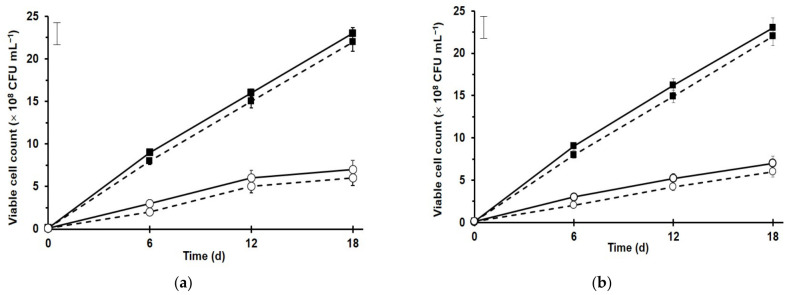
Evolution in the colony-forming units (CFUs) of *S. cerevisiae* strains Byosal HS1 (―) and IOC 18-2007 (- - -) over 18 days of fermentation in musts obtained from healthy (■) or Esca-proper-affected (○) *V. vinifera* cv Primitivo vines during 2023 (**a**) and 2024 (**b**). Each value represents the mean of two independent experiments with three replicates ± sd. The vertical bar indicates Fisher’s LSD at *p* < 0.05.

After 18 days in HV musts, the Byosal HS1 and IOC 18-2007 strains achieved 23 × 10^8^ and 22 × 10^8^ CFU mL^−1^, respectively (Figure 3). Compared to HVs, EpV musts reduced CFUs by 69.57 and 72.72% for Byosal HS1 and IOC 18-2007, respectively. Consistently, only *Saccharomyces* colonies were grown on YPDA and WL. Evolution in the growth of the two *S. cerevisiae* strains was strongly related to vine typology (Supplementary Table S2).

During the two years of must production, the fermentative vigour of the Byosal HS1 strain reached 5.988 ± 0.12 and 4.712 ± 0.13 g of CO_2_ per 100 mL in musts derived from berries collected from HVs and EpVs, respectively. Under identical conditions, the IOC 18-2007 strain achieved 6.121 ± 0.392 and 3.813 ± 0.509 g of CO_2_ per 100 mL of HV and EpV musts, respectively.

### 2.4. Wine Characterization

The enological characteristics of wines obtained from HV or EpV berries after fermentation with the two *S. cerevisiae* strains are detailed in Table 3. The examined wine attributes are linked to vine typology and *S. cerevisiae* strains (Supplementary Table S3). Wines from EpV musts showed higher sugar consumption and lower colour intensity, brilliance, alcoholic strength, and ethanol yield. Wines from HV musts showed higher colour hue, residual sugars, and titratable acidity (Table 3). After fermentation, in comparison to the initial musts (Table 2), the wine’s volatile acidity was reduced. A slight decline in the wine pH and L-malic acid content was recorded (Table 3). The wine produced from HVs using the IOC 18-2007 strain displayed higher volatile acidity (Table 3). No differences were recorded in the presence of lactic acid among the tested wines. Byosal HS1 produced the highest glycerol content in wines obtained with the two must typologies and the lowest total polyphenols in wines derived from EpV berries (Table 3). The wine made from the HV must confirms the absence of the three fungal secondary metabolites investigated. Pullulan, scytalone, and isosclerone were detected (Table 3) in the range of 3.878 ± 0.06–4.821 ± 0.08 ng L^−1^ in wine made from EpV musts.

## 3. Discussion

The EpV ‘Primitivo’ vines surveyed in this study displayed symptoms of cracks in the main trunk, tiger-striped leaves, sudden wilting on individual arms, branches, or shoots, black measles during the pre-veraison phenological stage, berry shrivelling and wilting, brown wood streaking, and white rot in branches or shoots. Wood microbiome analyses revealed *P. chlamydospora* and, to a lesser extent, *P. minimum* in tissues showing brown wood streaking, whereas *F. mediterranea* was detected in white-rotted tissues.

These findings align with previously described Esca-proper-affected vines [2,5,8,17,18,19,21,56,57,58,59]. Other fungi responsible for wood diseases in vines, such as species in the *Botryosphaeriaceae* and *Nectriaceae* families [60], have not been isolated. The absence of symptoms on leaves and berries during the 2018–2022 survey and in the wood tissues of branches and shoots, as well as the non-isolation of fungi that cause Esca-complex diseases on the vine, typifies the HV. From the surveyed and characterized vines, five HVs and EpVs were selected for grape harvesting and must production in 2023 and 2024. The five EpVs exhibiting symptomatic canopy in the 2023 vegetative season transitioned to asymptomatic in the 2024 growth period, validating the unpredictable annual Esca-proper symptom development [5,14,26,34,55]. This likewise confirms that in Esca-proper-affected plants, the absence of tiger stripes, apoplexy, berries’ black measles, shrivelling and wilting in a year does not indicate the absence of brown wood streaking, vascular occlusion and white rot in branches or shoots, or that the grapevine has eliminated the pathogens.

The values of total soluble solids (20.05–25.65 °Brix), total reducing sugars (172–250 g L^−1^), pH (2.98–3.42), titratable acidity (5.43–7.46 g L^−1^), tartaric acid (0.98–9.4 g L^−1^), L-malic acid (1.26–3.0 g L^−1^), and total polyphenols (316–2268 g L^−1^) available for different ‘Primitivo’ clones grown in Southern Italy [61,62,63,64,65,66] align with those of the HV must.

The lower level of total reducing sugars in the ‘Primitivo’ EpV must is consistent with the findings from ‘Trebbiano d’Abruzzo’ [51,52], ‘Cabernet Sauvignon’ [54], and ‘Sauvignon Blanc’ [67]. This decline may result from stomatal closure induced by xylem-invading pathogens [68,69,70,71,72,73], which reduces CO_2_ uptake, the photosynthesis rate, sugar synthesis, and translocation. Moreover, sugars could be diverted to the synthesis of plants’ secondary metabolites, reactive oxygen species (ROS), antimicrobial substances, enzymes, and tyloses involved in defence mechanisms [3,72,73,74,75]. The reduction in sugars in the EpV must is linked to the compromised functionality of tiger-striped leaves [52]. Furthermore, cell-wall-degrading enzymes and hydroxyl radicals produced by *P. minimum*, *P. chlamydospora*, and *F. mediterranea* [36,37,38,39] might influence sugar metabolism in leaves and grapes [76] and differentiate the musts obtained from HVs and EpVs in this study. The deleterious impacts of Esca-proper pathogens on vine physiology were also validated during the 2024 harvest, as the five selected EpVs displayed no tiger stripes, black measles, or apoplexy. In this context, it is reasonable to suggest that the presence and activity of the Esca-proper fungal consortium inhabiting the EpV wood might modify vine physiology [4,40,41,42,43,44,45,46,47,48,49] without symptom development. Additionally, the sugars available in the berries can be utilized as a respiratory substrate to produce malic acid, thereby increasing the total acidity [51,52,68,77,78]. The recorded data for titratable acidity, acetic acid, and L-malic acid do not support this metabolic evolution, showing lower levels in the EpV must, and do not align with the values in the musts considered by Calzarano et al. [51,52], Lorrain et al. [54], and Bruez et al. [67]. The different cultivars employed, the growth environment, and the so-called *terroir* effect may account for these differences. The wood-degradation activities of *P. chlamydospora*, *P. minimum*, and *F. mediterranea*, along with their metabolites (e.g., pullulan, scytalone, and isosclerone), enzymes (e.g., laccase), and by-products, could influence the composition of grapes and related musts. Citric and acetic acids in the must influence acidity and affect taste and mouthfeel. Citric acid content of up to 1.0 g L^−1^ enhances overall acidity, chelates metal ions, prevents browning, and exhibits antimicrobial properties. Acetic acid, the main contributor to volatile acidity in grape musts, results from the metabolism of acetic and lactic acid bacteria inhabiting the berries’ skin and acts as an indicator of spoilage [79]. The high levels of these two organic acids in HVs may be a result of the skin microbiome’s biodiversity, which typically includes lactic and acetic acid bacteria related to the quality and the sanitary condition of berries [79,80]. The volatile acidity levels in both HV and EpV musts are high for standard winemaking. The main source of volatile acidity before fermentation is the presence of grapes damaged by botrytis or sour rots, which contain *Acetobacter* spp. that convert sugars into acetic acid [79,80].

It should be noted that some of the differences observed between musts obtained from healthy and Esca-proper-affected vines may be related to differences in grape ripening. In the present study, HV musts showed higher total soluble solids and reducing sugars compared to EpV musts, suggesting a more developed technological maturity [79]. Consequently, the impact of vine sanitary status may be partially confounded by differences in the ripening stage, likely linked to alterations in canopy function and photosynthetic activity caused by Esca-proper mycobiota. Nonetheless, certain qualitative differences cannot be explained by ripening alone. The exclusive detection of pullulan, scytalone, and isosclerone in EpV musts suggests a direct influence of Esca-proper-related fungi on the berry’s composition. These metabolites, by-products of wood-colonizing pathogens [28,30,34], may impact yeast metabolism and fermentation performance [35]. Thus, while differences in ripening may play a minor role, the function of disease-related metabolites should be considered as another independent factor affecting the observed outcomes.

The total polyphenol amount in HV musts aligns with the findings from Calzarano et al. [51,52] and Lorrain et al. [54], where the must of Esca-infected vines was consistently higher than that of healthy vines. The production and accumulation of phenolic compounds serve as a defence strategy within the Plant Kingdom against both biotic and abiotic stresses [81]. Phenolics act as antioxidants to mitigate ROS impacts [82], chelate heavy metals [83], hinder pathogen spread, and affect cultivar susceptibility, pathogen attack, and host–pathogen interactions [84,85]. Phenolic compounds and their derivatives are specific components of grapes and musts, influenced by the grape ripening stage [86,87]. These compounds play a role in the aroma, colour, taste, bitterness, and astringency of grape berries, musts, and wines [88]. Vines affected by Esca-complex pathogens express defence-related genes linked to phenol pathways [76,89], whose products spread throughout the whole plant and are accumulated in the injured wood tissues [8,38,76,90] and in the leaves [91]. Degrading enzymes produced by *P. minimum*, *P. chlamydospora*, and *F. mediterranea* [37,76,84] affect phenolic metabolism in EpVs. In the infected plants, the net accumulation of phenolics is probably regulated by a balance that may occur, on one side, due to the production and accumulation of phenolics and, on the other side, from the pathogen’s tolerance to antifungal substances produced by the plant and the pathogen’s capacity to bioconvert the phenolics present [31,81].

Laccases (EC 1.10.3.2) are copper-containing proteins belonging to the class of blue oxidases. They oxidize different substances using oxygen as the final electron acceptor. Substrates oxidized by laccases include phenolics (e.g., phenols, polyphenols, and other related chemicals) and aromatic amines. Depending on oxygen availability, substrate concentration, and pH level, radicals formed by laccase activity produce quinones, dimers, polymers, and their mixtures. Laccases are produced by fungi, plants, and bacteria. Plants employ laccases in cell wall formation and in metabolic processes associated with defences [92,93]. Some laccases produced by plant-pathogenic fungi play roles in pigmentation, morphogenesis, delignification, and ROS generation [94]. Laccase produced in grapes by *Botrytis cinerea* (teleomorph *Botryotinia fuckeliana*), the causal agent of botrytis bunch rot in vineyards for wine and table grapes, influences wine organoleptic properties [95]. Laccase supports *F. mediterranea* in degrading lignin during wood decay and assists *P. chlamydospora* and *P. minimum* in detoxifying tannic acid, resveratrol, and other phenolic-derived phytoalexins [37]. The origin of laccase in EpV musts may be related to the infected plant, the berry-surface microbiome, and Esca-proper wood-colonizing fungi.

The detection of pullulan, scytalone, and isosclerone in the musts obtained from EpVs confirms that wood alterations, e.g., brown wood streaking and white rot, are associated with Esca-proper-related pathogens impacting the entire vine’s physiology [4,17,19,41], including grape and must composition [50,51,52,54]. These findings validate previous evidence on the presence of pullulan, scytalone, and isosclerone in the xylem sap collected during the bleeding phase, in the asymptomatic and tiger-striped leaves, in the rachis, and in berries [17,31,45,58,91]. The occurrence of *F. mediterranea* may also reduce these compounds [31,45,81], utilizing laccases to oxidize proanthocyanidins and anthocyanins [58], and lowering the concentration of phenolic compounds in wines obtained from EpVs.

From the winemaking point of view, soil geology, topography, climate and weather conditions, environmental stress factors, vineyard management practices, must composition, and vine cultivar influence yeast activity and govern alcoholic fermentation [79,96,97,98,99,100,101,102,103]. Starting with these general considerations, and to guarantee the predictability and reproducibility of the must-to-wine transformation, we opted for two commercial *S. cerevisiae* starters due to their enological performance in a synthetic must amended with pullulan or a mix of scytalone and isosclerone [35].

In the examined fresh Primitivo wines, colour intensity, hue, and brilliance were significantly affected by the *S. cerevisiae* strains and the different fermented musts. The enological parameters recorded for wine produced by fermenting must juice from HVs and EpVs are consistent with those reported in the literature [61,62,63,64,65,66] regarding alcoholic strength (11.5–15.22% *v*/*v*), pH (3.03–3.66), volatile acidity (0.1–3.2 g L^−1^), L-malic acid (0.38–2.37 g L^−1^), lactic acid (0.26–2.1 g L^−1^), titratable acidity (0.5–3.05 g L^−1^), and total polyphenols (824.33–3794 mg L^−1^).

Even without suitable experiments, Byosal HS1 and IOC 18-2007, at the end of fermentation, utilized an average of 99.83% of sugars in the EV must, in line with sugar utilization in synthetic musts and synthetic musts with pullulan [35]. When the two strains fermented the EpV must, only an average of 75.79% of the initial sugar content was consumed. This consumption agrees with the sugar utilization in synthetic musts containing a scytalone and isosclerone mix [35].

Another parameter to emphasize is the glycerol amount, which affects the organoleptic properties of wine. This sugar alcohol is produced during glycerol–pyruvic fermentation at the start of alcoholic fermentation, depending on the yeast strain, temperature, and pH in the must [79]. Glycerol defines various attributes in the taste of wine, including oiliness, persistence, and mellowness in the mouth. The final content of glycerol in the Primitivo wines analyzed here was affected by the *S. cerevisiae* strains. Byosal HS1 resulted in the highest glycerol levels after fermentation of both must typologies. The least amount was derived from the activity of IOC 18-2007. The Primitivo wine produced in this work, using IOC 18-2007 and EpV musts, exhibited the lowest glycerol concentration. In accordance with the Crabtree effects, IOC 18-2007 shifts the transformation of sugars in glycerol–pyruvic fermentation to produce ethanol and stimulates other metabolic pathways [35,79].

In the fresh Primitivo wine herein analyzed, scytalone and pullulan levels are half of those found in the EpV must, while the isosclerone concentration rises. The exopolysaccharide pullulan could be degraded by the physical–chemical conditions during the fermentation process. The metabolic pathway triggered by *S. cerevisiae*, along with the fermentation conditions, may lead to a shift in scytalone and isosclerone levels. These two molecules, like other naphthalenone pentaketides, are involved in fungal melanin biosynthesis [104,105,106,107,108]. Scytalone acts as an intermediate, and isosclerone is a by-product of scytalone oxidation [31,105,106,107,108].

The two fermented must typologies also differentiate alcoholic strength and ethanol yield between the two *S. cerevisiae* strains due to initial sugar concentration, yeast growth, and by-products [79].

Among the enological parameters, the reduction in tartaric acid content in wine compared to the amount found in the must is noteworthy and surprising. The *S. cerevisiae* strains are unable to utilize tartaric acid [79], suggesting possible precipitation during the fermentative process associated with the EpV must composition.

The volatile acidity in the wines obtained here relates to both the substrate and the tested *S. cerevisiae* strains. This enological trait, commonly linked to acetic acid, is a key sensory component in wine taste and appreciation. It depends on physical factors [98,109], *S. cerevisiae* strains [109,110,111,112,113], must composition and pH [45,112]. Regarding this enological trait, an unusually high volatile acidity was noted in the wine produced from the HV must fermented with the IOC 18-2027 strain. This suggests an atypical fermentation outcome. No similar increases in volatile acidity were observed in the other fermentations, indicating that this behaviour might be specific to this treatment. Given the magnitude of this disparity, this outcome probably indicates a deviation from the general fermentation pattern observed in the current study. Therefore, this treatment should be interpreted carefully and was not considered representative for the comparison of HV and EpV wines in the overall understanding of the results.

The level of total polyphenols in the wines is strongly associated with must typology and *S. cerevisiae* strains. Phenolic substances play a key role in the quality of red wines, such as Primitivo. Grape cultivar, vineyard agronomical techniques, winemaking technologies, and the yeast employed in fermentation affect the polyphenol content and typology in red wine [79,88]. Starting with two distinct must types (HVs and EpVs) that have comparable total polyphenol contents (ranging from 378.101 ± 8.089 to 323.714 ± 8.345 mg L^−1^), the two tested *S. cerevisiae* strains differentiate the total polyphenols concentration in the wines. The lower polyphenol amount was reached with Byosal HS1 using an EpV must, while the highest polyphenol content was obtained in the other must–strain combinations. The research on ‘Trebbiano d’Abruzzo’, ‘Cabernet Sauvignon’, and ‘Sauvignon Blanc’ only supports the trend between Byosal HS1 and EpV musts [51,52,54,67]. These differences could be explained by the fermentative and metabolic abilities of the two tested strains, the production or presence of anthocyanin-hydrolysing enzymes or polysaccharide-binding polyphenols, and the absorption of anthocyanins onto the yeast cell wall [79,88,111]. Additionally, *S. cerevisiae* employs the shikimate pathway to synthesize the aromatic ring from glucose via 3-deoxy-D-arabino-heptulosonate-7-phosphate synthase (EC 4.1.2.15; DAHP synthase), which increases the presence of polyphenolic metabolites in wine in standard wine-making conditions [107].

## 4. Materials and Methods

### 4.1. Vine Selection

A ‘Primitivo’ vineyard (50 vines) grafted onto 34EM rootstock established in 2002 at the collection field of the Council for Agricultural Research and Economics, Research Center Viticulture and Enology (CREA-VE) in the countryside of Rutigliano (Bari, southern Italy, 125 m asl) was used in this study. The vines were trained by the ‘Tendone’ system (1.25 m and 2.50 m spacings within and between rows, respectively) and cultivated with irrigation in alkaline clay soil. The vineyard was managed manually according to the common practices for the cultivar in the region. Starting from the 2018 vegetative season (ranging from pea-sized berries to technological maturity phenological stages), all vines were inspected for crack symptoms in the main trunk, tiger stripes, sudden wilting, black measles, berry shrivelling, and wilting development. Vines showing no visible symptoms were likewise annotated. From the 50 vines, branches (2 per plant) and shoots (5 per vine) were collected during the 2022 pruning operations (last week of November). These samples were deprived of cortical tissues and split longitudinally. Slices (1.5–2 cm in length) were surface sterilized for 2 min with 70% (*v*/*v*) ethanol, soaked for 1–2 min in sodium hypochlorite solution (3% active chlorine), rinsed three times with sterile distilled water, dried on a sterile paper towel, and then cut into small pieces. From each vine, fragments were taken from diseased areas (e.g., brown or rooted wood) or collected randomly from different points of unaltered wood tissues. A total of 1500 pieces were seeded onto 90 mm diam Petri dishes (five per plate, three plates per plant). Malt extract (2%) agar (MEA) amended with 0.25% Chloramphenicol (MEAC; 750 fragments) and MEA added with 0.25% Benomyl (MEAB; 750 fragments) served as semi-selective isolation media to detect phaeotracheomycotic and basidiomycete fungi, respectively. Inoculated plates were incubated at 25 ± 1 °C in the dark for 4–7 weeks. Emerging mycelia were cultured on Potato Dextrose Agar (PDA) plates. The isolates’ identification was based on colony morphology and molecular features using the primer pairs Pch1-Pch2 [114], T1-Bt2b [115], and ITS5-ITS4 [21] for *P. chlamydospora*, *P. minimum*, and *F. mediterranea*, respectively. The isolation frequency (IF) of each fungal taxon was calculated as IF = 100 × (N_i_/N_t_), where N_i_ indicates the number of wood fragments from which the fungus was isolated, and N_t_ is the total number of seeded pieces (N_t_ = 750 for each isolation medium). Among the 50 ‘Primitivo’ vines, after the five-year external symptoms survey and wood microbiome analyses, ten plants were selected, excluding the most external plants. Five symptomless plants that did not show any foliar symptoms and wood alterations, or Esca-associated fungi, were designed as healthy vines (HVs). Considering the frequency of symptom occurrences throughout 2018–2022, without the vines with complete apoplexy, five vines were selected as Esca-proper-affected vines (EpVs). The EpVs are characterized by brown wood streaking and white rot inside the trunk, along with crack symptoms on the main trunk, tiger stripes, sudden wilting, black measles, berry shrivelling, and wilting development during the survey period.

### 4.2. Must Production and Characterization

In addition to the absence of independent biological replicates, grapes from five vines per group (HVs and EPVs) were pooled to obtain representative musts for each condition. This approach minimizes variability related to individual plants and focuses on the effect of the different sanitary status of the vines. Berries from the selected HVs and EpVs were manually harvested at their technological maturity in September 2023 and 2024. The collected grape bunches were pooled within each group (HV and EpV) before crushing to obtain representative composite musts for each phytosanitary status. Consequently, a single must was produced for each condition and used for the successive fermentation trials. The grape bunches were mechanically crushed with a Polsinelli Enologia S.r.l. (Isola del Liri, Italy) destemmer crusher and subsequently pressed with a Polsinelli Enologia S.r.l. vertical press. The must yield was standardized at 75% (*v*/*w*). In the musts, total soluble solids were detected with an MA871 digital refractometer (Milwaukee Instruments, Inc., Rocky Mount, NC, USA) at 20 °C and expressed in degrees Brix. The pH was measured using a 5011T Hach glass Electrode with a Crison BasiC20 pH metre (Hach Lange, Torre Realia, Spain). Titratable acidity was determined with 0.1 N NaOH using bromothymol blue as an indicator. Colour analysis was carried out following the reference protocol outlined by Ribéreau-Gayon et al. [79] and by the International Organisation of Vine and Wine (OIV) [116] using a UV/Vis spectrophotometer Mod 8453 (Agilent Technologies Italy S.p.A., Milan, Italy) to read the absorbances (A) at 420 nm (yellow), 520 nm (red), and 620 nm (blue). The colour intensity (I) and hue (N; also labelled as shade) were calculated using the formulas I = A_420_ + A_520_ + A_620_ and N = A_420_/A_520_. Total sugars and tartaric, L-malic, lactic and acetic acids, volatile acidity, and total polyphenol concentrations were ascertained with a Hyperlab multiparametric analyser (Steroglass S.r.l., Perugia, Italy) according to the OIV enzymatic method [117]. Laccase activity was measured following the syringaldazine method [118] after pre-treatment with polyvinylpolypyrrolidone (PVPP; Sigma-Aldrich, a part of Merck Life Science S.r.l., Milano, Italy, code 38429) to strip out high levels of phenolic compounds that interfere with optical readings. Laccase from *Trametes versicolor* (Sigma-Aldrich) was used as a control. Because syringaldazine is highly specific to laccase over peroxidases, particular attention was given to pH, buffer composition, and substrate dissolution. A laccase calibration curve was prepared in the range of 0–100 U mL^−1^. Pullulan, scytalone, and isosclerone extraction and quantification were conducted as previously reported [29,31,35]. The two pentaketides were extracted using ethyl acetate at a 1:1 (*v*/*v*; must:solvent) ratio. The pullulan was precipitated with three volumes of cold absolute ethanol (15 h, −20 °C), collected by centrifugation (Thermo Scientific SL8R centrifuge; Thermo Fisher Scientific Inc., Waltham, MA, USA, at 8000 rpm, 20 min, 4 °C), and washed three times with 15 mL of cold methanol. Quantitative analysis was performed using Shimadzu Prominence LC-20 high-resolution liquid chromatography (HPLC) equipment with UV detector SPD-M20A, and LabSolutions software v5.82 (Shimadzu, Kyoto, Japan). A Waters (Waters S.p.A., Sesto San Giovanni, Italy) Nova-Pak C18 column (150 mm × 3.9 mm i.d., 4 μm particles) and a Nova-Pak C18 Guard Column (60A, 4 μm, 20 mm × 3.9 mm i.d.) were used, and the effluent was monitored at 280 nm for scytalone and isosclerone. A Waters 410 refractometer, along with a 1000 Å Nucleogel GFC column (300 × 7.7 mm i.d., Macherey-Nagel, Allentown, PA, USA), was used for pullulan analysis. Pullulan (P4516; Sigma-Aldrich), scytalone, and isosclerone samples were used as standards to calculate the calibration curve and to run co-injections with the analyzed samples. Determinations were performed on six repetitions. To avoid the negative effects on vinification of the microbiome (yeasts, lactic, propionic, acetic bacteria, and mould) present on the ‘Primitivo’ skin of the berries and available in the musts, and to prevent oxidation by the tyrosinase and laccase enzymes, musts from EpVs and HVs were amended with CX Potassium Metabisulphite E 224 (Corimpex Service S.r.l., Romans d’Isonzo, Italy) at 0.2 g L^−1^ [79,85] and stored in glass demijohns at 4 ± 1 °C until the use (48 h after vintage).

### 4.3. Fermentation Experiments

The *S. cerevisiae* commercial strains Byosal HS1™ (Esseco S.r.l., Divisione Enartis San Martino, Trecate, Italy) and IOC 18-2007™ (Institut OEnologique de Champagne, Reims, France) were used. The two strains were kept as stock cultures at 4 ± 1 °C on Yeast Peptone Dextrose Agar (YPDA: 1% yeast extract, 2% peptone, 2% dextrose, 2% technical agar N 3 from Oxoid, part of Thermo Fisher Scientific-Microbiology, Hampshire, UK) for short-term storage [35,116,119]. Fermentation trials were performed in two experiments with three technical replicates using the same must for each sanitary condition. It should be noted that independent biological replicates at the vinification level were not included in this experimental design, and therefore, the fermentation results reflect the behaviour of the representative composite musts rather than variability among independent biological units. Erlenmeyer flasks (500 mL nominal vol) topped with a cotton cap were filled with 200 mL of must, inoculated with 1 × 10^6^ cells mL^−1^, and incubated as stationary cultures at 20 ± 1 °C. Population dynamics were monitored every 6 d for 18 d by plating five independent dilutions on YPDA. The presence of undesirable *Pichia*, *Candida*, or *Brettanomyces* species was ascertained on Wallerstein Laboratory Nutrient Agar (WL; VWR International S.r.l., Milan, Italy) plates examined after 72 h of incubation at 25 ± 1 °C in the dark. The experiment was conducted at least twice, with three replicates. The fermentative vigour was investigated according to the OIV-OENO 370-2012 method [119] in 500 mL flasks sealed with a Müller trap, containing 100 mL of must with an initial sugar concentration of 250 gL^−1^ (adjusted adding a 1:1 *w*/*w* glucose:fructose solution), yeast inoculum of 1 × 10^8^ cells mL^−1^, and expressed as grams of CO_2_ (calculated as 2.5 × ∆_weight_) evolved by 100 mL of must during 3 d at 25 ± 1 °C under stationary cultures. Two experiments were performed using the same must for each vine typology with three technical replicates per strain.

### 4.4. Wine Characterization

Each fermentative unit was stopped when a residual sugar concentration of <2 g L^−1^ was detected in the Erlenmeyer flasks containing HV musts. Classic enological parameters of wine were measured immediately after the fermentation process was completed. Residual sugars, tartaric acid, L-malic acid, acetic acid (volatile acidity), total polyphenols, pullulan, scytalone, and isosclerone concentrations, I and N, were measured as aforementioned for the musts. Wine colour brilliance (WB) was calculated as follows [79]: WB = 100 × [1 − (A_420nm_ + A_620nm_)/(2 × A_520nm_)]. Wine pH was measured using a Crison Basic 20 pH-metre and a glass XS Foodtrode Electrode (Mettler Toledo, Milan, Italy). Glycerol and lactic acid were detected using a Hyperlab wine analyser. Alcoholic strength was determined with a Gibertini Digital Distilling Unit (Gibertini Elettronic, Milano, Italy), following the OIV-MA-AS312-01 method [116] and OIV-MA-AS1-13 guidelines [117]. Considering the maximum theoretical yield of ethanol from sugar as 0.51 g g^−1^, the ethanol yield was calculated as the ratio between the maximum concentration of ethanol produced (g L^−1^) and the amount of sugar consumed (g L^−1^). All determinations were performed on two experiments with three technical replicates per strain and typology of must.

### 4.5. Statistical Analysis

For each parameter, the mean and the standard deviation (sd) were calculated during the experiments. Following verification of the normality of distribution and the homoscedasticity of variance using the Shapiro–Wilk test and Levene’s test, respectively, data were analyzed for vine typology (HV and EpV), collection years (2023 and 2024), fermentation experiments (experiment 1 and experiment 2 performed using the same must for each vine typology with three technical replicates), and their interactions. The analysis of variance (ANOVA) with 95% confidence levels and Fisher’s least significant difference (LSD) was performed using SAS software version 9.0 for Windows.

## 5. Conclusions

The ‘Primitivo’ vines affected by Esca proper, associated with the presence of *P. chlamydospora*, *P. minimum*, and *F. mediterranea* in the woody tissues, alter plant physiology and influence musts both quantitatively and qualitatively. As expected, the musts obtained from EpVs show lower colour intensity and hue, total soluble solids, reducing sugars, titratable and volatile acidity, and tartaric, citric, and L-malic acid, as well as total polyphenol content. To the best of our knowledge, this is the first report of higher laccase activity and the occurrence of pullulan, scytalone, and isosclerone in musts produced by Esca-proper-affected vines. The modified makeup of EpV musts differentiate the growth and the enological performance of Byosal HS1 and IOC 18-2007, the two tested *S. cerevisiae* strains, and the ensuing fresh Primitivo wines. Wine made from the musts of EpVs exhibits low colour intensity and brilliance, reduced sugar consumption, lower alcoholic strength, ethanol yield, titratable acidity, L-malic and lactic acid levels, and total polyphenols, but has increased residual sugar. Slight differences influence pH, volatile acidity, and glycerol concentrations. Statistical analysis supports that the enological characteristics of musts were not affected by the sampling years but were closely linked to vine typology. The cause of the changes might be associated with the presence of laccase, pullulan, scytalone, and isosclerone related to the metabolic activity of *P. chlamydospora*, *P. minimum*, and *F. mediterranea* within the infected woody tissues. The physical–chemical conditions developed during the fermentation process may degrade pullulan and aid in the oxidation of scytalone to isosclerone, altering their relative concentrations.

The results obtained so far on ‘Primitivo’ vines indicate that Esca-proper-affected vines negatively impact grape composition and fermentation performance, leading to wines with reduced enological quality traits. However, it should be emphasized that some of these differences might be associated with differences in grape ripening, potentially related to the Esca-proper infection. Finally, the winemaking results reported here should be considered as preliminary due to the absence of biological replication during fermentation.

## 6. Limitations

This study identifies several limitations that need to be recognized.

First, the winemaking experiments were carried out using pooled musts obtained from multiple vines for each condition (HVs and EpVs), and fermentation trials were performed with technical replicates rather than independent biological replicates at the vinification stage. As a result, the statistical inference of wine-related parameters is limited, and the outcomes should be considered exploratory.

Second, differences in grape composition between HVs and EpVs suggest that the two must typologies were not at the same ripening. This may have partially influenced the observed differences in fermentation behaviour and final wine composition.

Third, the occurrence of an anomalous increase in volatile acidity in one treatment (HV must fermented with IOC 18-2007) further highlights the variability inherent in microvinification experiments and the necessity of careful interpretation.

These limitations do not compromise the overall interpretation of the current findings; instead, they highlight the necessity for additional studies aimed at confirming the results under replicated vinification conditions and at better elucidating the specific role of metabolites produced by Esca-proper fungal consortia in shaping wine composition.

## Figures and Tables

**Table 1:** Isolation frequency (%) ^1^ of fungi obtained on Malt Extract Agar Chloramphenicol (MEAC) or Malt Extract Agar Benomyl (MEAB) from unaltered (UWT), browned (BWT), or rooted (RWT) wood tissue collected in January 2023 by 50 *V. vinifera* ‘Primitivo’ vines with (present) or without (absent) tiger-stripe symptoms.

Fungal Isolate	Tiger-Stripe Symptoms:					
	Absent		Present			
	MEAC	MEAB	MEAC		MEAB	
	UWT	UWT	BWT	RWT	BWT	RWT
*Phaeomoniella chlamydospora*	0	0	46	6	0	0
*Phaeoacremonium minimum*	0	0	24	2	0	0
*Fomitiporia mediterranea*	0	0	0	0	64	66
*Paecilomyces* sp.	0	0	4	2	0	0
*Alternaria* sp.	4	0	2	3	0	0
*Penicillium* sp.	4	0	6	2	4	4
Other	0	0	6	6	8	8
No isolation	92	100	12	14	24	22

^1^ Isolation frequency (IF) of each fungal taxon was calculated as IF = 100 × (N_i_/N_t_), where N_i_ and N_t_ are the number of wood fragments from which the fungus was isolated and the total number of seeded pieces, respectively. N_t_ = 750 for each isolation medium.

**Table 2:** Characteristics of musts obtained from healthy or Esca-proper-affected *V. vinifera* cv Primitivo vines during 2023 and 2024 ^1^.

Parameters	Vines			
	Healthy		Esca-Proper-Affected	
	2023	2024	2023	2024
Vines (No)	5	5	5	5
Bunches (No/kg)	12/16 ± 0.14 a	12/16.5 ± 0.2 a	12/11 ± 0.2 a	12/10.8 ± 0.10 a
Colour intensity	1.449 ± 0.015 b	1.468 ± 0.009 b	0.918 ± 0.009 a	0.916 ± 0.017 a
Colour hue	2.049 ± 0.035 b	2.081 ± 0.035 b	1.475 ± 0.035 a	1.438 ± 0.011 a
Total soluble solids (°Brix)	24.517 ± 0.115 b	24.583 ± 0.041 b	19.275 ± 0.117 a	18.951 ± 0.015 a
Total reducing sugars (g L^−1^)	243.667 ± 0.307 b	244.167 ± 0.296 b	187.512 ± 0.307 a	185.816 ± 0.187 a
pH	3.23 ± 0.04 a	3.28 ± 0.03 a	3.36 ± 0.04 a	3.34 ± 0.01 a
Titratable acidity (g L^−1^)	6.843 ± 0.163 b	6.925 ± 0.116 b	4.593 ± 0.163 a	4.575 ± 0.187 a
Tartaric acid (g L^−1^)	1.205 ± 0.059 b	1.230 ± 0.059 b	1.165 ± 0.06 a	1.151 ± 0.173 a
L-malic acid (g L^−1^)	1.688 ± 0.020 b	1.709 ± 0.002 b	0.752 ± 0.021 a	0.717 ± 0.043 a
Citric acid (g L^−1^)	0.515 ± 0.015 b	0.539 ± 0.015 b	0.277 ± 0.015 a	0.259 ± 0.031 a
Volatile acidity as acetic acid (g L^−1^)	9.125 ± 0.022 b	9.104 ± 0.023 b	6.125 ± 0.022 a	6.247 ± 0.097 a
Total polyphenols (mg L^−1^)	378.101 ± 8.089 b	371.772 ± 8.089 b	331.965 ± 8.089 a	323.714 ± 8.345 a
Laccase activity (U min^−1^)	3.251 ± 0.011 a	3.123 ± 0.018 a	179.864 ± 0.016 b	169.758 ± 0.058 b
Pullulan (ng L^−1^)	0.0 a	0.0 a	8.113 ± 0.099 b	7.212 ± 0.011 b
Scytalone (ng L^−1^)	0.0 a	0.0 a	10.135 ± 0.012 b	11.352 ± 0.041 b
Isosclerone (ng L^−1^)	0.0 a	0.0 a	2.121 ± 0.013 b	2.428 ± 0.071 b

^1^ Each data point represents the mean of two independent experiments with three replicates ± sd. For each parameter, values marked with the same letter are not statistically significant according to Fisher’s LSD at *p* < 0.05.

**Table 3:** Enological characteristics ^1^ of wines obtained after the fermentation (18 d, 20 ± 1 °C, stationary cultures, in the dark) of musts extracted during 2023 and 2024 from healthy or Esca-proper-affected *V. vinifera* ‘Primitivo’ berries with *S. cerevisiae* strains Byosal HS1 or IOC 18-2007.

Parameters	Healthy				Esca-Proper-Affected			
	Byosal HS1		IOC 18-2007		Byosal HS1		IOC 18-2007	
	2023	2024	2023	2024	2023	2024	2023	2024
Colour intensity	9.841 ± 0.05 b	9.859 ± 0.05 b	5.11 ± 0.04 b	5.13 ± 0.05 b	3.81 ± 0.05 a	3.43 ± 0.04 a	4.31 ± 0.04 a	4.335 ± 0.05 a
Colour hue	0.660 ± 0.04 a	0.665 ± 0.01 a	0.787 ± 0.03 a	0.791 ± 0.09 a	0.972 ± 0.04 b	0.987 ± 0.01 b	1.191 ± 0.04 b	1.197 ± 0.01 b
Colour brilliance	63.286 ± 0.07 b	63.688 ± 0.07 b	62.051 ± 0.08 b	62.445 ± 0.07 b	48.801 ± 0.47 a	49.114 ± 0.61 a	33.321 ± 0.77 a	33.539 ± 0.04 a
Reducing sugars (g L^−1^)	0.021 ± 0.09 a	0.023 ± 0.02 a	0.333 ± 0.01 a	0.335 ± 0.05 a	49.333 ± 0.09 b	49.635 ± 0.05 b	15.001 ± 0.01 b	15.093 ± 0.01 b
Sugar consumption (g L^−1^)	243.232 ± 0.03 b	243.739 ± 0.02 b	242.912 ± 0.03 b	243.407 ± 0.09 b	138.119 ± 0.34 a	138.972 ± 0.17 a	172.521 ± 0.03 a	173.584 ± 0.01
Alcoholic strength (% *v*/*v*)	13.512 ± 0.07 b	13.593 ± 0.01 b	12.467 ± 0.03 b	12.541 ± 0.05 b	10.467 ± 0.07 a	10.529 ± 0.01 a	11.033 ± 0.07 a	11.098 ± 0.08 a
Ethanol yield	0.751 ± 0.07 c	0.753 ± 0.09 c	0.641 ± 0.023 b	0.643 ± 0.08 b	0.561 ± 0.06 a	0.562 ± 0.07 a	0.511 ± 0.06 a	0.517 ± 0.01 a
pH	2.94 ± 0.08 a	2.96 ± 0.02 a	3.05 ± 0.035 a	3.07 ± 0.04 a	3.087 ± 0.09 a	3.109 ± 0.04 a	3.147 ± 0.09 a	3.176 ± 0.13 a
Volatile acidity (g L^−1^) ^2^	0.111 ± 0.05 a	0.114 ± 0.06 a	1.251 ± 0.02 b	1.159 ± 0.01 b	0.157 ± 0.03 a	0.156 ± 0.03 a	0.253 ± 0.03 a	0.268 ± 0.07 a
Titratable acidity (g L^−1^) ^3^	0.871 ± 0.07 a	0.882 ± 0.02 a	0.807 ± 0.07 a	0.817 ± 0.05 a	0.787 ± 0.07 a	0.797 ± 0.01 a	0.741 ± 0.01 a	0.751 ± 0.01 a
L-malic acid (g L^−1^)	1.161 ± 0.09 b	1.172 ± 0.07 b	1.341 ± 0.03 b	1.343 ± 0.01 b	0.657 ± 0.08 a	0.654 ± 0.06 a	0.687 ± 0.08 a	0.698 ± 0.06 a
Lactic acid (g L^−1^)	0.073 ± 0.03 a	0.084 ± 0.01 a	0.041 ± 0.03 a	0.048 ± 0.03 a	0.011 ± 0.01 a	0.014 ± 0.02 a	0.006 ± 0.02 a	0.0073 ± 0.02 a
Total polyphenols (mg L^−1^)	1818.333 ± 0.14 c	1822.548 ± 0.19 c	1715.671 ± 0.15 c	1719.245 ± 0.17 c	473.334 ± 0.13 a	469.297 ± 0.11 a	1064.667 ± 0.02 b	1074.252 ± 0.12 b
Glycerol (g L^−1^)	1.172 ± 0.07 b	1.186 ± 0.02 b	0.207 ± 0.03 a	0.215 ± 0.08 a	1.191 ± 0.07 b	1.201 ± 0.06 b	0.191 ± 0.07 a	0.197 ± 0.09 a
Pullulan (ng L^−1^)	0 a	0 a	0 a	0 a	3.878 ± 0.06 b	3.904 ± 0.04 b	3.928 ± 0.06 b	3.954 ± 0.05 b
Scytalone (ng L^−1^)	0 a	0 a	0 a	0 a	4.011 ± 0.07 b	4.038 ± 0.05 b	4.021 ± 0.06 b	4.147 ± 0.05 b
Isosclerone (ng L^−1^)	0 a	0 a	0 a	0 a	4.821 ± 0.08 b	4.852 ± 0.06 b	3.985 ± 0.08 b	4.011 ± 0.05 b

^1^ Each data point represents the average of two experiments with three replicates ± sd; in rows, values with the same letter are not statistically significant according to Fisher’s LSD at *p* < 0.05. ^2^ Expressed as acetic acid. ^3^ Defined as tartaric acid.

## Data Availability

The original contributions presented in this study are included in the article/supplementary material. Further inquiries can be directed to the corresponding author.

## Supplementary Materials

The following supporting information can be downloaded at: https://www.mdpi.com/article/10.3390/plants15142187/s1.

## Abbreviations

The following abbreviations are used in this manuscript.

BWT	Browned Wood Tissue
CFU	Colony-Forming Unit
DAHP	3-deoxy-D-arabino-heptulosonate-7-phosphate
EC	Enzyme Commission
EpV	Esca-Proper-Affected Vine
GLSD	Grapevine Leaf Stripe Disease
HPLC	High-Resolution Liquid Chromatography
HV	Healthy Vine
I	Colour Intensity
IF	Isolation Frequency
LSD	Least Significant Difference
MEAB	Malt Extract Agar Benomyl
MEAC	Malt Extract Agar Chloramphenicol
N	Colour Hue or Colour Shade
N_i_	Number of Wood Fragments from which the Fungus was Isolated
N_t_	Number of the Total Seeded Wood Pieces
PDA	Potato Dextrose Agar
ROS	Reactive Oxygen Species
RWT	Rooted Wood Tissue
sd	Standard deviation
UWT	Unaltered Wood Tissue
WL	Wallerstein Laboratory Nutrient Agar
YPDA	Yeast Peptone Dextrose Agar
