# Supplementary material for: Effects of Musts from Esca-Proper-Affected ‘Primitivo’ Vines on Two Commercial Saccharomyces cerevisiae Strains
Source: Plants (Basel). 2026 Jul 16;15(14):2187. doi: 10.3390/plants15142187 (PMC13417375; doi:10.3390/plants15142187)
Supplement: Supplementary file 1 [file plants-15-02187-s001.zip › plants-4369071-supplementary.pdf]

Supplementary Table S1. Results of the analysis of variance (ANOVA) considering effects of sampling year (SY), vine typology (VT), experiments (Ex), and their interactions on bunches number (BN) and quantity (BQ), colour intensity (CI) and hue (CH), total soluble solids (TSS), total reducing sugars (TRS), pH (pH), titratable acidity (TitA), tartaric acid (TA), L-malic (MA) and citric (CA) acid, volatile acidity (Vac), total polyphenols (TP), laccase activity (Lac), pullulan (Pul), scytalone (Scy) and isosclerone (Iso)

Source of variation	df	F values[a]															
		BN	BQ	CI	CH	TSS	TRS	pH	TitA	TA	MA	CA	Vac	TP	Lac	Pul	Scy	Iso
SY	1	-	1.84	4.16	9.27	0.23	2.14	0.81	0.23	0.05	0.75	0.43	0.31	4.87	356.04	43.96	22.94	8.70
VT	1	-	2341.84*	17567.9*	16637.4*	2128.13*	2725.95*	26.18*	1192.04*	6.69**	13381.9*	3000.89*	1034.3	181.59*	6025545*	70578.1*	69498.5*	1909.88*
Ex	1	-	1.11	0.03	7.68	0.53	6.52	4.84	13.13	5.29	0.68	12.54	0.24	0.27	1.12	23.24	0.73	0.09
SY×VT	1	-	10.02**	6.40**	21.40**	17.78**	11.67**	3.90**	0.56**	0.72**	11.29**	19.87**	0.62	0.08	5092.10**	243.96**	222.94**	8.70**
SY×Ex	1	-	0.02	0.67	0.07	0.00	1.35	0.01	0.00	0.19	1.53	1.94	0.00	0.53	0.81	0.00	0.00	0.00
VT×Ex	1	-	0.02	0.67	0.07	0.00	1.35	0.00	0.00	0.19	1.53	1.94	0.00	0.53	0.80	23.24	0.73	0.09
SY×VT×Ex	1	-	0.02	0.67	0.07	0.00	1.35	0.01	0.00	0.19	1.53	1.94	0.00	0.53	0.81	0.00	0.00	0.00

[a] * and ** indicate $p = 0.01$ and $p = 0.05$, respectively.

Supplementary Table S2. Results of the analysis of variance (ANOVA) considering effects of sampling year (SY), vine typology (VT), *S. cerevisiae* strains (SC), experiment and their interactions on colony forming unit (CFU)

Source of variation	df	F values[a] CFU
SY	1	0.00
VT	1	74.09*
SC	1	20.65*
Ex	1	0.03
SY×VT	1	0.05
SY×SC	1	0.00
VT×SC	1	0.00
SY×Ex	1	0.00
VT×Ex	1	0.01
SC×Ex	1	0.00
SY×VT×SC	1	0.00
SY×VT×Ex	1	0.00
SY×SC×Ex	1	0.00
VT×SC×Ex	1	0.00
SY×VT×SC×Ex	1	0.00

[a] * indicate $p = 0.01$.

Supplementary Table S3. Results of the analysis of variance (ANOVA) considering effects of sampling year (SY), vine typology (VT), *S. cerevisiae* strains (SC) and their interactions on Colour intensity (I), hue (H), and brilliance (B), reducing sugars (ReS), sugar consumption (CoS), alcoholic strength (AS), ethanol yield (EtY), pH (pH), volatile acidity (VolAc), titratable acidity (TitAc), L-malic (MA) and lactic acid (LA), total polyphenols (TP), glycerol (Gly), pullulan (Pul), scytalone (Scy) and isosclerone (Iso)

Source of variation	df	F values[a]																
		I	H	B	ReS	CoS	AS	EtY	pH	VolAc	TitAc	MA	LA	TP	Gly	Pull	Scy	Iso
SY	1	0.05	0.04	0.06	0.02	0.06	0.06	0.00	0.07	0.01	0.07	0.00	0.03	0.00	0.04	0.03	0.03	0.03
VT	1	471.43*	201.24*	251.26*	2515.64*	291.64*	58.82*	52.22*	1.67	517.28*	0.06	515.87*	35.87*	78.81*	950.29*	2596.69*	2590.96*	2525.85*
SC	1	179.79*	46.75*	33.76*	658.26*	11.00*	0.67**	13.05*	0.81	863.76*	2.18	16.97*	5.22**	39.08*	826.89*	0.11	0.00	22.75*
Ex	1	2.74	1.04	1.59	0.99	11.02*	2.87	1.95	1.06	0.88	2.84	0.06	1.31	10.37*	1.21	0.67	0.66	0.61
SY×VT	1	0.00	0.00	0.00	0.02	5.32	0.00	0.00	0.00	0.02	0.00	0.00	0.00	0.00	0.00	0.03	0.03	0.03
SY×SC	1	0.00	0.00	0.00	0.01	0.00	0.00	0.00	0.00	0.00	0.00	0.00	0.00	0.00	0.01	0.00	0.00	0.00
SY×Ex		2.27	0.66	2.10	0.99	0.00	2.54	1.45	1.54	0.64	0.88	1.83	0.00	2.05	0.90	1.09	1.09	1.07
VT×SC	1	275.79*	3.09	24.52*	682.63*	2.50	7.13**	1.63	0.07	541.10*	4.05	7.42**	2.32	651.95*	0.26	0.11	0.00	22.75*
VT×Ex	1	0.47	0.53	0.09	0.96	11.44	0.02	0.02	0.00	0.36	0.00	0.18	0.01	0.03	0.01	0.67	0.66	0.61
SC×Ex	1	0.00	0.37	0.01	0.27	0.12	0.00	0.00	0.00	0.29	0.00	0.01	0.00	0.02	0.35	0.00	0.00	0.01
SY×VT×SC	1	0.00	0.00	0.00	0.01	0.00	0.00	0.00	0.00	0.00	0.00	0.00	0.00	0.01	0.00	0.00	0.00	0.00
SY×VT×Ex	1	0.19	0.55	0.09	0.96	0.00	0.02	0.02	0.00	0.23	0.00	0.18	0.01	0.03	0.00	1.09	1.09	1.07
VT×SC×Ex	1	0.01	0.19	0.01	0.28	0.12	0.00	0.00	0.00	0.09	0.00	0.00	0.00	0.27	0.00	0.00	0.00	0.01
SY×VT×SC×Ex	2	0.10	0.27	0.01	0.28	0.00	0.00	0.00	0.00	0.29	0.00	0.00	0.00	0.14	0.17	0.00	0.00	0.01

[a] * and ** indicate $p = 0.01$ and $p = 0.05$, respectively.
